# Concluding Observations

**Published:** 1855-08

**Authors:** 


### CONCLUDING OBSERVATIONS.

While Cholera prevails, no marked change should be made as to the general habits of a regular temperate life—as long as the person feels entirely well—but the moment the great premonitory symptom is observed even in a slight degree, to wit, *an indefinable uncomfortableness in the belly, inclining to rest*, then an instantaneous change should be made from physical activity to bodily rest—from mental activity to mental relaxation—from the habitual use of wines, or malt, or other alcoholic drinks to total abstinence—from everything of the kind; using ice or ice-water as a substitute, or cold spring water, a few swallows only in any twenty minutes; but if ice is to be had, and there is thirst, it may be eaten continuously from morning until night.

Whatever may have been the diet before, it should be changed at once to tea and toast, or cold bread and butter, with plain meat, salted or fresh, whichever is relished most—I mean that these changes should be made on the first appearance of *belly-uncomfortableness*, and if in six or eight hours you are not decidedly better, send for a physician. If you are better, continue your own treatment until the feeling in the belly has entirely disappeared and you have a desire to walk about, and experience a decided relief in doing so.

If you have over two (or three at most) passages within twenty-four hours, do not make an experiment on your life by taking even a calomel pill, simple as it is, unless it be wholly impracticable to obtain a physician within three or four hours.

